# Depression, anxiety, suicidal risks and clinical correlates among migrant Egyptian physicians a cross-sectional study

**DOI:** 10.1007/s44192-026-00439-y

**Published:** 2026-04-13

**Authors:** Mervat Said, Eman Mandour, Eman Fathey ElSemary, Hibatallah M. Fawzy, Eman Fauad

**Affiliations:** 1 Psychiatry Department, Faculty of Medicine, Zagazig University, P. O. Box 44519, Zagazig, Egypt; 2 Public Health and Community Medicine Department, Zagazig University, Zagazig, Egypt

**Keywords:** Migrant physicians, Anxiety, Depression, Suicide

## Abstract

**Background:**

Moving to a new country for work can be incredibly stressful, especially for physicians who already face high-pressure jobs. This study explores symptoms of depression and anxiety, along with self-reported suicidal ideation and attempts, among Egyptian doctors working abroad.

**Methods:**

A cross-sectional online survey of 400 Egyptian immigrant physicians in Arab Gulf/Western nations was conducted via Facebook professional groups using comprehensive consecutive sampling. Data were collected using a questionnaire covering sociodemographic, clinical, and immigration-related factors, and history of suicidal ideation/ attempts, and the Hospital Anxiety and Depression Scale (HADS) was used to assess the severity of anxiety and depressive symptoms.

**Results:**

About 16% of doctors reported moderate to severe depression. Women were 2.5 times more likely to experience it than men (aOR = 2.5, 95% CI: 1.4–4.4). Nearly 40% struggled with significant anxiety, especially those working more than 8 h a day. 6.3% of doctors had suicidal thoughts, and almost 5% had attempted suicide. Younger physicians were found to be fourfold more likely to have suicidal risk compared to their older counterparts (aOR = 4.2, 95% CI: 1.9–9.0), while physicians who reported barriers to accessing mental health services had a 2.9-fold increased risk of suicidal risk (aOR = 2.9, 95% CI: 1.2–3.9). The reported barriers included a lack of insurance coverage, fear of losing their jobs, and stigma.

**Conclusion:**

Egyptian migrant physicians face significant mental health burdens, exacerbated by being younger, long working hours, past mental health issues, and systemic barriers to care. Urgent interventions must address healthcare access, occupational safeguards, and culturally sensitive support to mitigate suicide risk and improve well-being in this critical workforce.

## Background

Physician migration represents a global phenomenon with profound personal and professional implications [1, 2]. Worldwide, migration commonly occurs from less-developed to more-developed countries [3]. Egyptian doctors increasingly seek opportunities abroad—primarily in the Gulf and Western countries—driven by systemic challenges including inadequate compensation, strenuous work environments, and limited professional autonomy in their home country. While such migration offers career advancement, the journey carries a significant psychological impact [4].

Despite growing recognition of mental health challenges among migrant populations globally [5, 6], the unique burdens facing *physician* migrants remain critically understudied. Emergent research confirms that relocation itself amplifies vulnerability to psychological distress, with immigrants facing elevated risks of depression, anxiety, and identity conflicts [7, 8].

Prior work identifies high rates of depression, substance use, and sleep disorders in general migrant workers [9, 10]; therefore, physicians practicing abroad confront unique psychological burdens as they navigate licensing complexities, credential reevaluation, and high-pressure clinical contexts—all while adapting to unfamiliar cultural settings.

Review of literature highlights the scarcity of studies on physician migrants’ mental health and suicide risk, a population where untreated distress jeopardizes both clinician well-being and patient safety [11]. Our research bridges this evidence gap by examining depression, anxiety, and suicidality among Egyptian physician expatriates, quantifying prevalence and identifying modifiable risk factors to shed light on rapid-needed interventions.

## Materials and methods

### Study design and participants

We conducted a cross-sectional online survey targeting Egyptian physicians practicing abroad. Participants were recruited via country-specific, physician-exclusive Facebook groups across six destination countries: the Gulf Cooperation Council (GCC) countries (Kingdom of Saudi Arabia [KSA], United Arab Emirates [UAE], Kuwait, and Oman) and Western regions (United States [USA], United Kingdom [UK], Germany).

### Sample size and design

Initially, 441 subjects participated in this study. Following screening, 41 respondents were excluded due to incomplete response (defined as failure to complete the core survey items, including either key demographic questions and /or the scale questions, *n* = 29) or failure to meet the inclusion criteria (*n* = 12), yielding a final sample of 400 migrant physicians. Inclusion criteria were: licensed physicians of Egyptian origin, of any sex, actively practicing abroad for at least one year, and aged between 30 and 60 years. To ensure Egyptian nationality, eligibility was determined via a two-step screening process within the survey: (1) Initial screening questions verified Egyptian nationality and a primary medical qualification from Egypt. (2) Subsequent questions confirmed current medical licensure and practice abroad.

Exclusion criteria included severe comorbid physical illnesses (such as renal or hepatic failure, or malignancy), current substance use disorders, and physicians who had returned to practice in Egypt within the past year. To ensure confidentiality and minimize response bias, the survey was conducted anonymously. No personally identifiable information was collected. Participants were assured of data anonymity in the digital consent form and instructions, which emphasized the independence and privacy of their responses. Data was stored on a secure, password-protected server accessible only to the research team.

A minimum target sample of 385 participants was calculated using Open Epi software (Version 3.01), based on a conservative depression/anxiety prevalence estimate of 50% derived from physician population studies [12, 13]. Our use of a 50% (*p* = 0.5) prevalence estimate in the sample size calculation was a deliberate and conservative methodological choice. This parameter was selected not as an expected outcome but to ensure the study would have adequate power and precision across the broad spectrum of prevalence rates reported in physician mental health literature. This calculation ensured 80% statistical power to detect significant effects within a 95% confidence interval and ± 5% margin of error. We employed comprehensive consecutive sampling to inclusively enroll all eligible Egyptian migrant physicians practicing in target destinations during a six-month recruitment window (September 2019–February 2020).

### Data collection tool

Data were collected via a structured electronic questionnaire assessing four domains: (1) Sociodemographic characteristics (age, sex, marital status, pre-migration Egyptian residence [urban/rural], current living arrangement “alone /with family”); (2) Clinical history (personal/family/past psychiatric history, current medical comorbidities, substance use disorders excluding nicotine)(; (3) Occupational and migration factors (professional title, specialty, daily work hours [≤ 8 vs. >8], weekly workdays, annual vacation days, and mental healthcare access barriers [dichotomized yes/no; open-text specification for reasons); and (4) Psychometric assessments, including the Hospital Anxiety and Depression Scale (HADS) [14], (An Arabic-validated form by Moussa et al. (2016) [15] was administered.). It is a 14-item self-report tool (7 anxiety + 7 depression items; 4-point Likert scales [0–3]; subscale range 0–21) with scores ≥ 11 indicating clinically significant symptoms. —alongside direct screening for suicide risk through two items assessing 1-month suicidal ideation (“Have you considered ending your life in the past month?“) and attempts with yes/ no answers.

### Statistical analysis

The collected data underwent digital processing and statistical analysis using IBM SPSS Statistics (Version 27.0). Categorical variables were summarized as frequency counts with proportional percentages, with between-group differences assessed via chi-square (χ²) tests. Continuous measures were reported as means ± standard deviations (SD), with independent samples t-tests comparing normally distributed variables across two groups. The charts were created using Microsoft Word for graphical presentation. Univariate logistic regression initially screened variable associations, with significant predictors (*p* < 0.05) advancing to multivariable models controlling for confounders. Results were expressed as adjusted odds ratios (aOR) with 95% confidence intervals (CI). Model calibration was verified through Hosmer-Lemeshow goodness-of-fit testing. Statistical significance thresholds were defined as: *p* > 0.05 (non-significant), *p* ≤ 0.05 (significant), and *p* ≤ 0.001 (highly significant).

## Results

The socio-demographic and clinical characteristics of the 400 participants are summarized in Table 1. The mean age of the subjects was 39.4 years (± 6.3 SD). Most of them were male (62.3%, *n* = 249), married (87.8%, *n* = 351), residing in urban areas (91.3%, *n* = 365), and living with their family (74.5%, *n* = 298). Registrars constituted the largest professional group (58.0%, *n* = 232), followed by residents (18.5%, *n* = 74) and consultants (14.5%, *n* = 58). The most common specialties were Medicine (33.5%, *n* = 134) and Surgery (26.8%, *n* = 107). A minority reported a positive family history of psychiatric illness (19.7%, *n* = 92), a past psychiatric history (24.7%, *n* = 115), or a present medical history (26.0%, *n* = 104).

**Table 1:** The sociodemographic and clinical variables among the studied group

Variable	Immigrant (*n* = 400)
Age	
Mean ± **SD	39.4 ± 6.3
Gender:	
Female	151 (37.8%)
Male	249 (62.3%)
Social status:	
Married	351 (87.8%)
Single/Divorced/Widowed	49 (12.3%)
Residence:	
Rural	35 (8.8%)
Urban	365 (91.3%)
Living Arrangement:	
Alone	102 (25.5%)
With family	298 (74.5%)
Clinical title:	
Consultant	58 (14.5%)
**GP	36 (9%)
Registrar	232 (58%)
Resident	74 (18.5%)
Specialty:	
Medicine:	134 (33.5%)
Surgery:	107 (26.8%)
**ER:	11 (2.8%)
**ICU:	38 (9.5%)
**GP:	15 (3.8%)
Pediatrics:	50 (12.5%)
Academic:	1 (0.3%)
Others:	44 (11%)
Family history of psychiatric illness	
No	374 (80.3%)
Yes	92 (19.7%)
Past history of psychiatric illness	
No	351 (75.3%)
Yes	115 (24.7%)
Present medical history:	
No	296 (74%)
Yes	104 (26%)

**GP General Practitioner, *ER* Emergency Room, *ICU* Intensive Care Unit, *SD* Standard Deviation

Concerning the work-related patterns and mental healthcare barriers among immigrant physicians (*N* = 400), the data showed that the mean vacation days per year is 33.4 (SD = 9.6). Workload patterns revealed that 327 participants (81.8%) exceeded 8 daily working hours, while 371 (92.8%) worked more than 4 days weekly. Regarding mental healthcare access, 178 physicians (44.5%) identified significant barriers to service utilization.

**Fig. 1:**
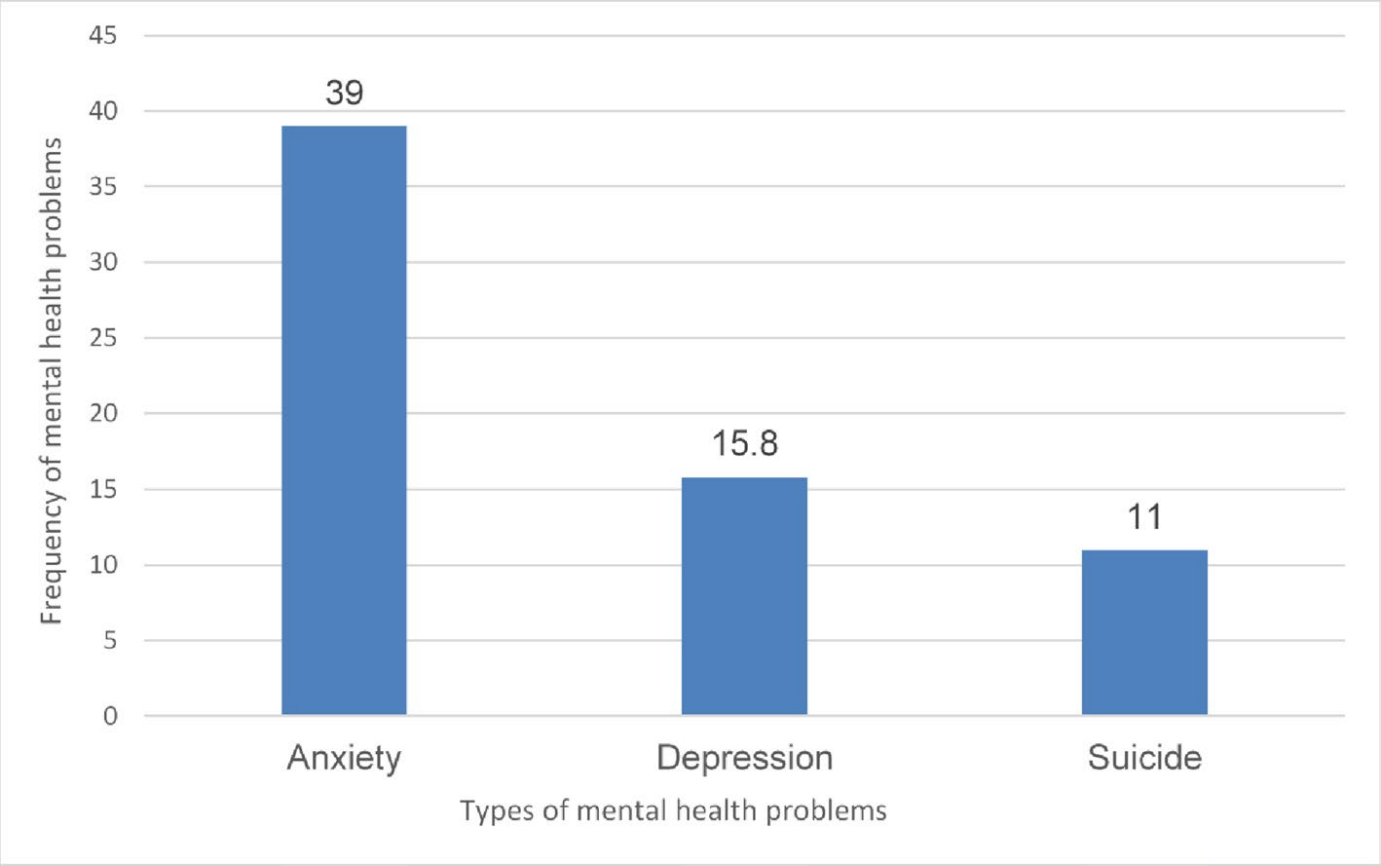
Bar chart showing the frequency of mental health problems among migrant physicians

Regarding the frequency of mental health problems among the migrant physicians, our results showed that moderate-to-severe anxiety affected 39% (*n* = 156) of participants, while 15.8% (*n* = 65) reported moderate-to-severe depression levels. Notably, 11% (*n* = 44) exhibited suicidal risk, comprising 6.3% (*n* = 25) with active suicidal ideation and 4.7% (*n* = 19) with suicide attempts during the last month, as shown in Fig. 1.

### Anxiety correlates

For the anxiety group, bivariate analyses examining demographic correlates (moderate-to-severe vs. normal-to-mild) revealed no statistically significant associations with age, sex, residence, living arrangement, clinical title, or medical specialty (all *p* > 0.05). Descriptive comparisons showed that the anxiety group (*n* = 156; mean age 38.9 ± 6.0 years; 56.4% female) and the non-anxiety group (*n* = 244; mean age 39.7 ± 6.5 years; 34.0% female) had similar age distributions (*p* = 0.226) and sex distributions (*p* = 0.054).

Notably, intensive care specialists represented 12.8% of the anxiety group versus 7.4% in the non-anxiety group—the largest proportional difference among specialties, though t did not reach statistical significance.

Variables related to clinical history factors demonstrated significant associations with the level of anxiety. Participants with moderate-to-severe anxiety were more than twice as likely to report a past psychiatric history compared to those without anxiety (21.8% vs. 9.4%; χ²=10.8, *p* = 0.001). However, there was no statistically significant relationship between significant anxiety level and positive family history of mental disorders (23.1% vs. 15.6%, *p* = 0.059)/nor present medical history among anxious participants (30.8% vs. 23.0%, *p* = 0.082).

**Table 2:** Relationship between anxiety and work-related conditions among the IMMIGRANT studied group

Variable	Without anxiety (*n* = 244)	With anxiety (*n* = 156)	*P*-value
Vacation days:			
Mean ± SD	33.7 ± 9.9	33.01 ± 9.2	0.472
Working hours/day			
≤ 8 h	208 (85.2%)	119 (76.3%)	0.02*
> 8 h	36 (14.8%)	37 (23.7%)	
Working days/ week			
≤ 4 days	14 (5.7%)	15 (9.6%)	0.144
> 4 days	230 (94.3%)	141 (90.4%)	
Clinical title:			
Consultant	42 (17.2%)	16 (10.3%)	0.234
GP	22 (9%)	14 (9%)	
Register	139 (57%)	93 (59.6%)	
Resident	41 (16.8%)	33 (21.2%)	
Specialty:			
Medicine:	89 (36.5%)	45 (28.8%)	0.190
Surgery:	70 (28.7%)	37 (23.7%)	
ER:	6 (2.5%)	5 (3.2%)	
ICU:	18 (7.4%)	20 (12.8%)	
GP:	10 (4.1%)	5 (3.2%)	
Pediatrics:	29 (11.9%)	21 (13.5%)	
Academic:	1 (0.4%)	0 (0%)	
Others:	21 (8.6%)	23 (14.7%)	
Barriers to use the mental health services:			
No	160 (65.6%)	62 (39.7%)	< 0.001**
Yes	84 (34.4%)	94 (60.3%)	

*P value of < 0.05 indicates a statistically significant result

**P value of < 0.001 indicates a highly significant result

As regards work-related factors, our results revealed significant associations with anxiety status (Table 2). Physicians with moderate-to-severe anxiety were more likely to work > 8 h daily (23.7% vs. 14.8%; χ²=5.46, *p* = 0.02) and nearly twice as likely to report barriers to mental health services (60.3% vs. 34.4%; χ²=25.2, *p* < 0.001) compared to those without anxiety.

**Table 3:** Regression analysis for anxiety’s predicting factors among immigrant physicians

Variable	B	Wald	OR (95%CI)	*P*-value
Past history of psychiatric disorder:	0.884	8.414	**2.4 (1.3–4.4)**	0.004 *
More than 8 h working per day:	0.478	3.016	1.6 (0.9–2.7)	0.082
Barriers to use mental health services:	1.022	22.51	**2.7 (1.8–4.2)**	< 0.001**

*P value of < 0.05 indicates a statistically significant result

**P value of < 0.001 indicates a highly significant result

Multivariable regression (Table 3) identified two significant predictors of moderate-to-severe anxiety among immigrant physicians. Barriers to mental health services emerged as the strongest predictor (OR = 2.7, 95%CI:1.8–4.2, *p* < 0.001). Past psychiatric history also demonstrated significant predictive value (OR = 2.4, 95%CI:1.3–4.4, *p* = 0.004). While extended work hours (> 8 h/day) showed a clinically relevant association (OR = 1.6, 95%CI:0.9–2.7), this did not reach statistical significance (*p* = 0.082).

### Depression correlates

Results of our research showed that moderate-to-severe depression (vs. normal-to-mild) showed significant demographic and clinical correlates. Female physicians had over 2.5 times higher depression prevalence (57.1% vs. 34.1%; χ²=11.2, *p* = 0.001). Clinical history revealed even stronger associations: those with depression were nearly three times more likely to report a history of mental illness (28.6% vs. 11.6%; χ²=14.9, *p* < 0.001) and twice as likely to have current medical conditions (41.3% vs. 23.1%; χ²=8.9, *p* = 0.003).

Work-related correlates of depression revealed one significant association: physicians with moderate-to-severe depression reported substantially higher barriers to mental health services compared to non-depressed counterparts (68.3% vs. 40.1%; χ²=17.2, *p* < 0.001). On the other hand, no significant differences emerged for Vacation days (31.7 ± 10.1 vs. 33.7 ± 9.5 days; t = 1.57, *p* = 0.119), proportion working > 8 h daily (77.8% vs. 82.5%; *p* = 0.374), or Weekly working patterns (> 4 days/week: 93.7% vs. 92.6%; *p* = 0.117). Notably, depressed physicians averaged fewer vacation days (31.7 vs. 33.7) and showed higher rates of extended work hours (22.2% working ≤ 8 h/day vs. 17.5%), though these differences were not statistically significant. Moreover, there was no significant relationship between the level of depression and clinical title or specialty.

**Table 4:** Regression analysis for depression’s predicting factors among the immigrant physicians

Variable	B	Wald	OR (95%CI)	*P*-value
Gender:	0.945	11.45	**2.5 (1.4–4.4)**	0.001 **
Past history of psychiatric disorder:	0.829	5.448	**2.2 (1.1–4.5)**	0.02 *
Present history of medical condition	0.665	4.572	**1.9 (1.1–3.5)**	0.03 *
Barriers to use mental health services:	1.074	12.63	**2.9 (1.6–5.2)**	< 0.001**

*P value of < 0.05 indicates a statistically significant result

**P value of < 0.001 indicates a highly significant result

Multivariable logistic regression (Table 4) identified four independent predictors of depression among immigrant physicians. Barriers to mental health services emerged as the strongest predictor (OR = 2.9, 95%CI:1.6–5.2, *p* < 0.001), nearly tripling depression odds. Female gender significantly increased depression risk (OR = 2.5, 95%CI:1.4–4.4, *p* = 0.001), as did past psychiatric history **(**OR = 2.2, 95%CI:1.1–4.5, *p* = 0.02). Current medical conditions also demonstrated significant predictive value (OR = 1.9, 95%CI:1.1–3.5, *p* = 0.03).

### Correlates of suicide

Significant demographic differences emerged between physicians with suicidal risk (ideation/attempts) and their non-suicidal counterparts. The suicidal group was younger (36.6 ± 6.1 vs. 39.7 ± 6.3 years; t = 3.15, *p* = 0.002) and had a higher representation of non-married individuals (i.e., Single/Divorced/Widowed) (22.7% vs. 11.0%; χ²=5.48, *p* = 0.02). Additionally, suicidal risk was strongly associated with clinical title.

Residents were more likely to screen positive for suicidal risk compared to attending physicians (29.5% vs. 17.1%; *p* < 0.05). Significant associations were also observed between specialty and suicidal risk, with higher prevalence among G.P.s (15.9% vs. 8.1%), Surgeons (36.4% vs. 25.6%), and ICU physicians (18.2% vs. 8.4%) compared to their respective reference groups (*p* = 0.04). No significant associations were found for gender, residence type, or living arrangement (all *p* > 0.05).

Clinical history factors demonstrated statistical associations with suicidal risk. Physicians with suicidal risk were nearly twice as likely to report positive family history of mental illness (31.8% vs. 16.9%; χ²=6.15, *p* = 0.01) and 2.6 times more likely to have past psychiatric history (31.8% vs. 12.1%; χ²=14.2, *p* < 0.001) compared to the non-suicidal group. In contrast, current medical conditions showed no significant association with suicidal risk (27.3% vs. 25.8%; *p* = 0.838).

Work-related factors, including clinical title, the specialty, and mental healthcare barriers, showed a significant association with suicidal compared to the non-suicidal group (*p* = 0.01; *p* = 0.04; *p* = 0.007). No significant differences for vacation days (32.9 ± 11.5 vs. 33.5 ± 9.4 days; *p* = 0.736), extended daily work hours (> 8 h: 25.0% vs. 17.4%; *p* = 0.219) or weekly working patterns (> 4 days/week: 86.4% vs. 93.5%; *p* = 0.083).

**Table 5:** Relationship between suicidal risk and frequency of anxiety and depression among immigrant physicians

Variable	Without suicidal ideation (*n* = 356)	With suicidal ideation (*n* = 44)	*P*-value
Anxiety:			
Absent:	229 (64.3%)	15 (34.1%)	< 0.001**
Present:	127 (35.7%)	29 (65.9%)	
Depression:			
Absent:	306 (86%)	31 (70.5%)	0.008*
Present:	50 (14%)	113 (29.5%)	

*P value of < 0.05 indicates a statistically significant result

**P value of < 0.001 indicates a highly significant result

Significant links were observed between suicidal risk and psychiatric symptoms (Table 5). Physicians reporting suicidal risk were more likely to exhibit moderate-to-severe anxiety/depression compared to those without such risk (65.9% vs. 35.7%; χ²=15.2, *p* < 0.001; and 29.5% vs. 14.0%; χ²=7.1, *p* = 0.008, respectively).

**Table 6:** Regression analysis for predicting factors of suicide among the studied group

Variable	B	Wald	OR (95%CI)	*P*-value
Age:	1.437	13.67	**4.2 (1.9-9)**	< 0.001*
Specialty (ICU):	0.064	0.898	1.1 (0.9–1.2)	0.343
Unmarried social status:	0.234	0.417	1.2 (0.6–2.5)	0.519
Last certificate (Bachelor’s degree, western certificate):	0.053	0.177	1.1 (0.8–1.3)	0.674
Clinical title (resident):	0.378	3.06	1.4 (0.9–2.2)	0.080
Family history of psychiatric disorder:	0.506	1.601	1.6 (0.7–3.6)	0.206
Past history of psychiatric disorder:	0.815	3.907	**2.2 (1-5.1)**	0.04 *
Barriers to use mental health services:	0.544	6.709	**2.9 (1.2–3.9)**	< 0.001**
Anxiety:	0.895	5.225	**2.4 (1.1–5.2)**	0.02*
Depression:	0.076	5.118	**1.9 (1.3–2.5)**	0.03*

*P value of < 0.05 indicates a statistically significant result

**P value of < 0.001 indicates a highly significant result

Multivariable logistic regression referred to four independent predictors of suicidal risk among the migrant physicians (Table 6). This table shows that age, the presence of a history of psychiatric illness, the presence of anxiety, depression, and the presence of barriers against the use of health services were found to be statistically significant predictors for suicidal risk among the immigrant physicians. Younger physicians were found to be fourfold more likely to have suicidal risk compared to their older counterparts (OR = 0.4.2 [1.9-9], *p* < 0.001). Those having a past history of psychiatric illness were found to be nearly two-fold more likely to have suicidal risk compared to those without a history (OR = 2.2 [1-5.1], *P* = 0.04). Also, subjects who experienced anxiety and depression were at nearly two-fold risk of suicide compared to those who didn’t experience either of them (OR = 2.4 [1.1–5.2], *P* = 0.02, OR = 1.9 [1.3–2.5], *P* = 0.03), respectively. Interestingly, migrant physicians who showed they had barriers against using health services were found to have nearly three times higher risk of suicidal risk compared to those who hadn’t (OR = 2.9 [1.2–3.9], *P* < 0.001).

**Fig. 2:**
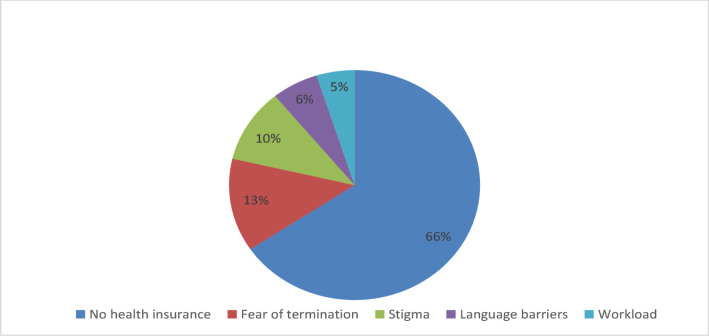
Pie diagram showing the frequency of barriers against seeking medical help among immigrant physicians

## Discussion

This research addresses a critical gap in understanding the psychological well-being of expatriate physicians, a population facing unique transnational stressors yet markedly understudied. In this cross-sectional study of 400 Egyptian migrant physicians, we assessed the frequency of depression and anxiety and examined correlates of suicidality.

### Frequency of mental health problems: depression and anxiety among migrant physicians

This study revealed substantial mental health burdens among Egyptian physician migrants, with moderate-to-severe depression affecting 15.8% and clinically significant anxiety present in 39% of participants. These findings are matched with global migrant health patterns: Foo et al. (2018) [16] documented nearly identical depression prevalence (15.6%) across 16,121 migrants spanning 20 nations, while Chen et al. (2019) [17]. observed higher depression rates (24.3%) among Chinese migrant participants. Contemporary meta-analytic evidence reinforces these epidemiological patterns, with Hasan et al. (2021) [8]. documenting aggregate depression (39.0%) and anxiety (27.3%) rates, aligning closely with our results.

Divergent prevalence patterns emerge when comparing our findings with prior migrant health studies. Hatch et al. (2016) [18]. documented lower depression (10.7%) and Generalized Anxiety Disorder GAD (6.9%) rates among their migrant cohort, while Adebayo et al. (2020) [19] reported minimal anxiety prevalence (5%). These methodological discrepancies likely reflect differences in study populations and psychometric instrumentation rather than true epidemiological variation.

Migration exerts profound and multifaceted impacts on mental health through interconnected stressors that extend through three temporal phases: pre-migration challenges—including socioeconomic precarity, disrupted social networks, trauma exposure, and political instability in origin countries; migration journey adversities—such as hazardous transit conditions, violence exposure, community separation, and resettlement uncertainty; and post-migration adjustments—featuring employment instability, social isolation, family separation distress, linguistic barriers, and cultural adaptation strains [20, 9, 21]. Physician migrants navigate additional profession-specific vulnerabilities wherein advanced education paradoxically amplifies psychological risk. Despite their qualifications, they frequently confront credential devaluation and language-mediated employment barriers [22]. While heightened educational attainment may foster critical consciousness of systemic inequities and elevate lifestyle expectations, creating cognitive dissonance when professional identities clash with occupational marginalization, ultimately generating distress pathways to mental disorders [23].

### Frequency of suicide among migrant physicians

Our findings reveal significant suicide vulnerability among Egyptian physician migrants: 11% exhibited suicide risk (6.3% active ideation; 4.7% attempts), aligning with established evidence of elevated suicidality in immigrant populations [6, 24]. This confirms the results of the previous work by Amir (2020) [25], which included a meta-analysis of 51 studies (*N* = 482,311 migrants) and reported 16% ideation and 6% attempt prevalence globally.

Acculturation stress—the psychological strain of adapting to new cultural environments—remains a critical suicide precipitant [26]. Again, for physicians, this is amplified by profession-specific stressors, systemic healthcare disparities, institutional stigma around help-seeking, and fear of professional consequences further heighten risk. These gaps create cascading vulnerabilities where untreated distress escalates to suicidality—a pattern acutely observed in our research, particularly among early-career physicians facing occupational instability.

Furthermore, pervasive “triple stigma” – encompassing personal shame, professional judgment, and institutional penalties – creates profound obstacles to care Contemporary data confirms that a majority (68%) of distressed physicians, particularly migrants, avoid treatment due to licensure fears, showing minimal improvement despite awareness efforts [27]. Deeply ingrained self-reliance norms, originating from training cultures emphasizing invulnerability, demonstrably delay intervention and mediate distress (37%), resulting in critically low help-seeking rates even among those experiencing suicidal ideation [28]. Additionally, traits like perfectionism, traditionally selected for medical excellence, become potent risk factors under chronic stress. When coupled with workload, they contribute to professional identity erosion – a disintegration of clinical self-worth that precipitates a crisis [29]. Collectively, these factors necessitate targeted interventions addressing lethal means safety, stigma reduction, cultural shifts towards help-seeking, and support systems mitigating perfectionism and identity [28–30].

**Fig. 3:**
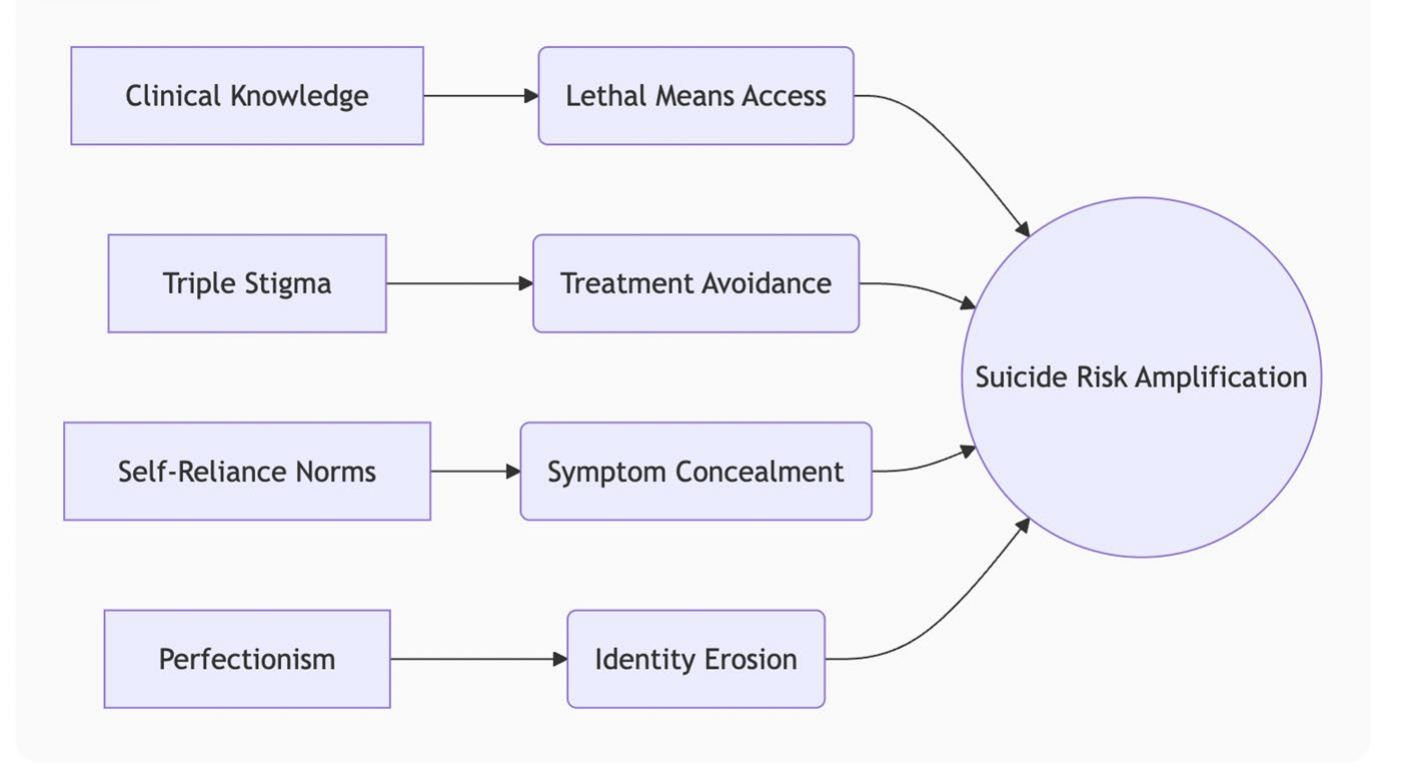
Interconnected factors that amplify the risk of suicide among migrant physicians

### Significant predictors of mental health problems and suicidal risk by regression analysis

1. Sociodemographic predictors

Regression analysis accounting for confounders identified female gender and comorbid medical conditions as significant predictors of depression; however, age was a highly significant factor for suicide among migrant physicians in our study. This aligns with extensive prior evidence demonstrating women’s heightened vulnerability to depression across populations, attributed to interrelated biological (e.g., genetic predisposition, hormonal fluctuations), sociocultural (e.g., gendered roles, adverse experiences), and psychological factors (e.g., differential stress reactivity, coping styles) [31, 32]. While these mechanisms likely contribute to observed disparities, the precise determinants remain incompletely elucidated [33, 32].

Our findings further corroborate established links between physical illness and depressive symptomatology. The frequent co-occurrence of medical conditions and major depressive disorder is increasingly understood through shared pathophysiological pathways, including common genetic variants, immune-inflammatory dysregulation, and illness-related psychosocial sequelae (e.g., altered social functioning) [34, 35]. These intersecting biological and environmental mechanisms substantiate the elevated depression burden observed in individuals managing chronic health challenges, consistent with recent epidemiological reports [35, 36].

One of the notable findings in this research was that younger Egyptian physician expatriates (< 40 years) showed a fourfold higher suicide risk than older colleagues (OR = 4.2 [1.9–9.0], *p* < 0.001). This aligns with global patterns: Lithuanian physicians under 45 exhibited elevated suicide risk due to career instability and inadequate coping [37]. Similarly, young Egyptian physicians face severe isolation and unsustainable workloads [38]. For expatriates, younger doctors’ limited cross-cultural adaptability exacerbates distress from workplace inequities or language barriers [39]. Their underdeveloped resilience and mentorship access heighten vulnerability during crises like COVID-19 [40]. Reinforcing this, physicians aged 30–50 experience 1.3× higher suicide mortality than the general population [41]. This convergence of evidence demands urgent, tailored support for early-career expatriates facing intersecting professional, financial, and acculturative stresses.

2. Clinical predictors

Our Research identified a pre-existing psychiatric history as an independent predictor of concurrent depression, anxiety, and suicidality among migrant physicians. This finding aligns with established evidence indicating prior depressive episodes significantly increase vulnerability to relapse or recurrence, particularly under adverse conditions, reflecting an ongoing neurobiological and psychological susceptibility [42, 43]. Furthermore, the likelihood of recurrence escalates with both the number of past episodes and the presence of comorbid anxiety, suggesting a cumulative pathophysiological burden [44, 45].

Our finding that a history of psychiatric illness was a strong predictor of current distress among migrant physicians must be understood within the context of the significant mental health burden present in the Egyptian medical workforce even before migration. Studies conducted in Egypt have shown alarmingly high rates of depression and anxiety among physicians, particularly during crises such as the COVID-19 pandemic [46,47]. This suggests that for some, migration may be an attempt to escape an already high-stress professional environment.

Critically, our research confirmed the profound link between active anxiety/depression and heightened suicide risk in this population. Affected participants exhibited nearly twice the odds of suicidality compared to unaffected peers (Anxiety: OR = 2.4, 95% CI [1.1–5.2], *p* = 0.02; Depression: OR = 1.9, 95% CI [1.3–2.5], *p* = 0.03). This corroborates the well-documented paradigm where psychiatric morbidity constitutes a primary driver of suicidal behavior, with epidemiological studies consistently reporting that > 90% of suicide attempters experience significant psychiatric illness [48–51]. Additionally, previous research found that both anxiety symptoms and depressive symptoms were independent risk factors for suicidal behaviors, which increase the risk for suicide [52–54].

Evidence indicates individuals with anxiety disorders frequently exhibit diminished distress tolerance and impaired emotion regulation capacities [55, 56]. This compromised ability to process and modulate negative affective states may heighten vulnerability to viewing suicidality as a perceived means of escape from psychological anguish [56]. Moreover, anxiety’s contribution to suicide risk demonstrates differential effects contingent on the severity of co-occurring depressive symptoms [57]. Consequently, comprehensive clinical assessment must account for these frequent psychiatric comorbidities to accurately evaluate risk profiles [54].

3. Work-related predictors

One of the striking findings in our research is that barriers to mental healthcare access constitute a robust independent predictor for depression, anxiety, and suicidality among migrant physicians. Notably, these structural obstacles emerged as one of the strongest predictors for both anxiety (OR = 2.7, 95%CI:1.8–4.2, *p* < 0.001) and depression (OR = 2.9, 95%CI:1.6–5.2, *p* < 0.001). Participants facing healthcare barriers demonstrated nearly threefold elevated suicide risk compared to unimpeded counterparts (OR = 2.9, 95%CI:1.2–3.9, *p* < 0.001). Reported barriers included insurance deficits (most prevalent), termination fears, stigma, language limitations, and time constraints due to workload, as showed Figs. 2, 3.

This aligns with established evidence documenting heightened psychiatric morbidity among immigrants encountering healthcare access challenges [58, 59, 8]. Comparable structural challenges—including financial constraints, linguistic barriers, insurance gaps, legal status complications, temporal limitations from intensive work schedules, cultural preferences for traditional healing, and geographic inaccessibility—commonly restrict healthcare utilization in migrant populations [60–63]. Furthermore, profound mental health stigma within many immigrant communities substantially interferes with service utilization [64–67].

Previous studies investigating barriers to help-seeking among distressed physicians have found that certain beliefs may prevent them from contacting health services [68, 69]. These reported barriers were primarily systemic (e.g., insurance deficits, fear of professional consequences). However, it is critical to acknowledge that help-seeking is also hindered by internalized factors, including beliefs prevalent within medical culture—such as viewing psychological distress as a sign of weakness or failure—which may be carried by physicians from their training environments and exacerbated by migration stress [69, 70]. Therefore, the construct of ‘barriers’ in this context encompasses a complex interaction between external systemic obstacles and internal, culturally-shaped beliefs, both of which must be addressed in supportive interventions. Our study shed light on the importance of access to mental health services for migrant physicians, which may minimize the mental burden of migration with better management of their mental health problems and ultimately reduce the suicidal risk and improve the quality of life.

Limitations: This study had some limitations. First, it is a cross-sectional study, which limits the establishment of a cause-and-effect relationship. Second, it depends on self-reporting scales and questions, which raises the possibility of bias. Third, stigma about having a mental disorder, especially among physicians, may lead to an underestimation of results. Fourth, recruitment was conducted exclusively via Facebook professional groups. While this provided efficient access to a widespread diaspora community, it may have introduced selection bias by underrepresenting physicians who are not active on social media or in online professional networks, potentially limiting the generalizability of the finding*s.* Fifth, while we assessed individual occupational factors (e.g., work hours, specialty), our study did not capture data on important institutional-level variables, such as the size and type of healthcare facility (e.g., large academic hospital vs. small private clinic) or the nature of the employer (e.g., government, private corporation, academic institution). Differences in workplace resources, organizational culture, contractual stability, and institutional support could significantly influence mental health outcomes and represent important confounding variables. Future studies should incorporate these structural factors to provide a more comprehensive understanding of the occupational determinants of well-being among migrant physicians. A further limitation of our analysis, the grouped participants across six host countries may obscure the unique effects of different cultural and systemic environments.

Although this study has many limitations, it also has many strengths. This study is one of the few, if not the only, to assess the mental health of migrating physicians. It included a large number of participants from immigrants to Arab and Western countries. It investigated as many aspects (demographic, clinical, social, and occupational) as possible of the risk factors of mental illness among Egyptian migrant physicians.

## Conclusions

Our findings highlight significant mental health challenges among physician migrants: anxiety shadows 39% of the Egyptian medical expatriate, depression affects 15.7%, and 11% face suicidal risk. Behind these numbers lie critical vulnerabilities—a prior history of psychiatric distress significantly heightens susceptibility to all three conditions, while female physicians and those managing medical illnesses are particularly prone to depression. Alarmingly, younger expatriates carry a fourfold greater suicide risk. Crucially, the greatest predictor of heightened mental health struggles is the presence of barriers to healthcare access. This research underscores an urgent moral and practical imperative: ensuring migrant doctors have unimpeded, compassionate access to mental health support is not merely beneficial—it is essential for safeguarding their well-being, enriching their quality of life, and ultimately preserving the skilled, resilient workforce upon which their host communities depend. Future research should prioritize longitudinal and cohort research to map the progression of mental health outcomes in migrant physicians, accounting for the complex interplay of demographic, biological, psychological, environmental, occupational, and social factors. This evidence base is crucial for subsequently assessing the impact and efficacy of tailored psychological and social support interventions.

## Data Availability

To support scientific transparency and collaboration, the datasets generated and analyzed during this study are maintained by the corresponding author and can be provided upon reasonable request.

## Abbreviations

ER: Emergency Room
GAD: Generalized Anxiety Disorder
GCC: Gulf Cooperation Council
GP: General Practitioner
HADS: Hospital Anxiety and Depression Scale
ICU: Intensive Care Unit
KSA: Kingdom of Saudi Arabia
UAE: United Arab Emirates
UK: United Kingdom
USA: United States
